# Evolutionary Relationships Between the Laccase Genes of Polyporales: Orthology-Based Classification of Laccase Isozymes and Functional Insight From *Trametes hirsuta*

**DOI:** 10.3389/fmicb.2019.00152

**Published:** 2019-02-06

**Authors:** Olga S. Savinova, Konstantin V. Moiseenko, Ekaterina A. Vavilova, Andrey M. Chulkin, Tatiana V. Fedorova, Tatiana V. Tyazhelova, Daria V. Vasina

**Affiliations:** ^1^Laboratory of Molecular Aspects of Biotransformations, A. N. Bach Institute of Biochemistry, Research Center of Biotechnology, Russian Academy of Sciences, Moscow, Russia; ^2^Laboratory of Gene Expression Optimization, A. N. Bach Institute of Biochemistry, Research Center of Biotechnology, Russian Academy of Sciences, Moscow, Russia

**Keywords:** laccases, polyporales, evolution, phylogenetics, gene-tree/species-tree reconciliation, isozymes

## Abstract

Laccase is one of the oldest known and intensively studied fungal enzymes capable of oxidizing recalcitrant lignin-resembling phenolic compounds. It is currently well established that fungal genomes almost always contain several non-allelic copies of laccase genes (laccase multigene families); nevertheless, many aspects of laccase multigenicity, for example, their precise biological functions or evolutionary relationships, are mostly unknown. Here, we present a detailed evolutionary analysis of the *sensu stricto* laccase genes (CAZy – AA1_1) from fungi of the Polyporales order. The conducted analysis provides a better understanding of the Polyporales laccase multigenicity and allows for the systemization of the individual features of different laccase isozymes. In addition, we provide a comparison of the biochemical and catalytic properties of the four laccase isozymes from *Trametes hirsuta* and suggest their functional diversification within the multigene family.

## Introduction

Wood-rotting fungi are crucial components of many terrestrial ecosystems. In addition to their fundamental roles in carbon balance, soil formation and forest regeneration, wood-rotting fungi provide a habitat for various organisms that support the biodiversity of our planet (Jönsson et al., 2008; Lonsdale et al., 2008; Marcot, 2017). Moreover, their ability to efficiently degrade lignocellulose biomass makes wood-rotting fungi, especially white rot fungi from the order Polyporales (Basidiomycota, Fungi), an attractive target for many biotechnological applications, such as the production of biofuel, biopulping and bioremediation (Pérez et al., 2002; Hatakka and Hammel, 2011; Fisher and Fong, 2014).

In the last decade, there has been an increasing interest in the evolution of wood-degrading fungi in general and their ligninolytic enzyme complex in particular (Binder et al., 2013; Riley et al., 2014; Justo et al., 2017). This notorious progress has been achieved in the case of fungal class II peroxidases (i.e., manganese, lignin and versatile peroxidases). Evolution of these enzymes was traced back to the Carboniferous geological period and associated with an appearance of different wood-decay strategies (i.e., white rot and brown rot) later in the Cretaceous (Floudas et al., 2012; Ruiz-Dueñas et al., 2013; Krah et al., 2018). Moreover, sequences of several ancestral peroxidase genes were inferred, and the corresponding enzymes were heterologously expressed and biochemically characterized (Semba et al., 2015; Ayuso-Fernández et al., 2017, 2018). Oddly enough, there are no recent reports devoted specifically to the evolution of laccases, which, along with peroxidases, are the main components of the extracellular enzymatic complex of white rot fungi.

Laccases (benzene diol:dioxygen oxidoreductases; EC 1.10.3.2; CAZy – AA1_1) are blue MCOs that catalyze single-electron abstractions from various phenolic and non-phenolic compounds, with a concomitant reduction of molecular oxygen to water (Baldrian, 2006; Morozova et al., 2007; Giardina et al., 2010; Mogharabi and Faramarzi, 2014). Broad substrate specificity and high stability make laccase a prospective enzyme for different biotechnological applications (Riva, 2006; Rodríguez Couto and Toca Herrera, 2006; Mate and Alcalde, 2017). It is currently well established that fungal genomes almost always contain several non-allelic copies of *sensu stricto* laccase-encoding genes – known as laccase multigene families; nevertheless, many aspects of evolution and functions of these families remain unknown.

It would be fair to say that, although many different laccase phylogenetic trees have been constructed with different purposes throughout the literature, at the moment, there is only a small number of works directly devoted to the evolution of laccases (Valderrama et al., 2003; Hoegger et al., 2006; Billette et al., 2011; Kües and Rühl, 2011). Moreover, all present works are very broad in their sampled taxa, incomplete in their inclusion of entire laccase multigene families and, consequently, are highly general in their conclusions. It was established that all Basidiomycete laccases form a single monophyletic branch – *sensu stricto* laccases – and many of them occasionally occur on tree leaves, according to the taxonomical associations of the corresponding fungal species (Hoegger et al., 2006; Kües and Rühl, 2011), suggesting both the absence of extensive gene conversion and a relatively small number of species-specific duplications. However, neither the precise subdivision of laccases into OGs nor the reconstruction of the most likely sequence of evolutionary events leading to the formation of these groups have ever been described.

In addition to the absence of a detailed description of the laccase evolutionary history, there is a lack of research devoted to the comprehensive biochemical characterization of different laccase isozymes from the same fungus. This gap in the literature hinders any attempts to resolve a long-standing question regarding which advantages allow for the ability to produce several laccase isozymes. Although several pieces of indirect evidence exist on the functional diversification of laccases in fungi, a consensus is yet to be reached (Kües and Rühl, 2011).

A lack of both a reliable picture of the laccase gene family evolution and the comparable biochemical data on different laccase isozymes present a substantial hindrance for understanding the possible range of laccase biological functions, with information scattered throughout the literature regarding their properties and determining new directions of studies of this undoubtedly biotechnologically promising enzyme.

In the present work, we aimed to reconstruct the evolutionary history of the *sensu stricto* laccase gene family (CAZy – AA1_1) from the wood-degrading fungi of the Polyporales order and to use the obtained evolutionary relationships for the orthology-based classification of the different laccase isozymes. For this purpose, we performed a gene-tree/species-tree reconciliation analysis for all of the 28 laccase multigene families and used the resulting phylogeny as a scaffold to systematize information about laccase isozymes with previously published properties. In addition, we provided a comparison of the biochemical and catalytic properties of the four laccase isozymes from *Trametes hirsuta*.

## Materials and Methods

### Fungal Strains

The following fungal strains were obtain from the Collection of the Komarov Botanical Institute (LEBIN), Russian Academy of Sciences (St. Petersburg): *Trametes ochracea* (LE-BIN 093), *Trametes gibbosa* (LE-BIN 1911), *Lenzites betulinus* (LE-BIN 2047), *Trametes hirsuta* (LE-BIN 072), *Phlebia chrysocreas* (LE-BIN 2009), *Steccherinum murashkinskyi* (LE-BIN 1963), *Antrodiella faginea* (LE-BIN 1998), *Antrodiella pallasii* (LE-BIN 2322), *Peniophora lycii* (LE-BIN 2142). Fungi were cultivated as described in Vasina et al. (2015).

To overcome the ambiguity in the fungal taxonomy and to exclude possible mistakes in the identification of the fungi, ribosomal ITS regions, ITS1 and ITS2, were sequenced for all fungi obtained from LEBIN, as described in the Section “DNA Extraction and ITS Sequencing”. Obtained ITS sequences were compared to those from fungi used in the PolyPEET project (Binder et al., 2013; Justo et al., 2017)^1^. Whenever sequence similarity of 95–100% was detected fungus from the LEBIN was regarded as the same species with fungus used in the PolyPEET project.

For heterologous expression of laccase isozymes *Penicillium canescens* PCA-10(niaD^-^) strain (Aleksenko et al., 1995) was used.

### DNA Extraction and ITS Sequencing

For DNA extraction, fungal mycelium was ground in liquid nitrogen, and total DNA was extracted using DNeasy Plant Mini Kit (Qiagen, United States), according to the manufacturer’s instructions.

ITS1 and ITS2 sequences were amplified using standard PCR primers: ITS1F (5^′^-CTTGGTCATTTAGAGGAAGTAA-3^′^) and ITS4B (5^′^-CAGGAGACTTGTACACGGTCCAG-3^′^). PCR amplification was performed using the Encyclo PCR kit (Evrogen, Russia) under the following conditions: 1 cycle of 5 min at 95 °C; 25 cycles of (1 min at 90°C, 1 min at 56°C, and 1 min at 72°C); 1 cycle of 10 min at 72°C. Obtained PCR products of ∼720 bp were purified from the agarose gel by the QIAquick Gel Extraction Kit (Qiagen, United States) and sequenced by the standard Sanger sequencing method.

### RNA Extraction and Rapid Amplification of cDNA Sequence Ends (RACE-PCR)

For RNA extraction, fungal mycelium was ground in liquid nitrogen, and total RNA was extracted using RNeasy Mini Kit (Qiagen, United States), according to the manufacturer’s instructions. The reverse transcription of total RNA to double stranded complementary DNA (cDNA) was performed using polyA specific primers and MINT cDNA kit (Evrogen, Russia), according to the manufacturer’s instructions.

Initial “middle” laccase fragments used in RACE-PCR primer design were obtained previously by the 454 pyrosequencing procedure as described in Moiseenko et al. (2016). All 5^′^/3^′^ RACE-PCR reactions were carried out according to the instructions in the Mint RACE cDNA amplification set (Evrogen, Russia). Obtained RACE-PCR products were resolved in 1.4% TAE agarose gel and specific bands were purified using QIAquick Gel Extraction Kit (Qiagen, United States). Sequencing of purified RACE-PCR products was done by the Sanger method.

The GenBank accession numbers of the obtained 39 sequences are placed in the Supplementary Table S1.

### Evolutionary Study of the Polyporales Sensu Stricto Laccases

#### Fungal Species and Species-Tree

For the current analysis, 28 fungal species of the Polyporales order were selected: from the **CorePol_Clade** – *Trametes villosa*, *Trametes versicolor*, *Trametes ochracea*, *Trametes sp.* AH28-2, *Trametes gibbosa*, *Lenzites betulinus*, *Trametes ijubarskyi*, *Trametes cingulata*, *Trametes hirsuta*, *Pycnoporus sanguineus*, *Pycnoporus coccineus*, *Pycnoporus cinnabarinus*, *Pycnoporus puniceus*, *Polyporus arcularius*, *Polyporus brumalis*, *Lentinus tigrinus*; from the Antrodia clade – *Fomitopsis pinicola*, *Wolfiporia cocos*; from the Phlebioid clade – *Phlebia brevispora*, *Phlebia chrysocreas*, *Phanerochaete carnosa*, *Phanerochaete chrysosporium*, *Phlebiopsis gigantea*, *Bjerkandera adusta* (the last four fungi formed a distinct Phanerochaecaceae subclade); and from the Residual Polyporoid clade – *Antrodiella faginea*, *Antrodiella pallasii*, *Steccherinum murashkinskyi*, *Cerrena unicolor*.

The species-tree topology was extracted from the phylogenies constructed under the **PolyPEET** project (Binder et al., 2013; Justo et al., 2017), and the main milestones on the evolutionary timeline of the Polyporales order were extracted from (Garcia-Sandoval et al., 2011; Floudas et al., 2012; Krah et al., 2018).

#### Collection of the Laccase Sequences

All laccase sequences used in this study (Supplementary Table S1) belong to the *sensu stricto* laccases of AA1_1 CAZy subclass and are of the three kinds: (1) sequences derived from the automatically annotated publicly available whole genomes (JGI and NCBI databases); (2) experimentally determined sequences from GeneBank databases; (3) sequences that were experimentally determined by the authors. The sequences experimentally determined by the authors were either obtained by the 454-pyrosequencing procedure described in (Moiseenko et al., 2016) or by the RACE-PCR procedure described in the Section “RNA Extraction and Rapid Amplification of cDNA Sequence Ends (RACE-PCR).” Whenever it was possible, automatic genome annotations was validated against the experimentally determined gene structures. As a result, 153 non-redundant laccase sequences from the selected fungi of the Polyporales order were collected. Additionally, 3 laccase sequences from *P. lycii* belonging to the Russulales order, which is distantly related to the Polyporales order and hence to all selected for the current investigation fungi, was obtained and used as an out-group during the laccase gene-tree reconstruction.

#### Preliminary Laccase Gene-Tree

To construct preliminary laccase gene-tree, codon-based multiple sequence alignment of the collected laccase nucleotide sequences was constructed using MUSCLE algorithm (Edgar, 2004; Larsson, 2014). Suitable nucleotide substitution model, GTRΓ + I, was determined using jModelTest2 software (Darriba et al., 2012) under the Akaike information criterion (AIC) criterion. Phylogenetic tree was reconstructed under the maximum likelihood criterion (ML) with RAxML-HPC BlackBox (8.2.10) program (Stamatakis, 2014) at the CIPRES Science Gateway (Miller et al., 2010)^2^.

#### Synteny-Aware Gene-Tree/Species-Tree Most Parsimonious Reconciliation and Final Laccase Gene-Tree

The preliminary laccase gene-tree was further reconciled with the fungal species-tree via the synteny-aware gene-tree/species-tree most parsimonious reconciliation procedure with simultaneous local rearrangements of the low supported branches. This methodology was previously theoretically described and algorithmically formalized in (Lafond et al., 2013a,b), Wu et al. (2013), and Noutahi et al. (2016); however, in our case just manual intervention was enough. The low supported branches on the initial gene-tree were manually rearranged (topologically constrained) in a manner that minimized the number of gene loss/duplication events and placed the most closely located on the chromosome genes in the closest possible topological proximity on the gene-tree.

The final laccase gene-tree was reconstructed with RAxML-HPC BlackBox (8.2.10) program (Stamatakis, 2014) at the CIPRES Science Gateway (Miller et al., 2010)^2^ under the GTRΓ + I substitution model and defined above topological constrains. The statistical equivalence between initial and modified gene-tree topologies were established by the likelihood ratio test. The bootstrap vales were assigned with SumTrees package (Sukumaran and Holder, 2010) based on the bootstrap replicates obtained during the preliminary laccase gene-tree construction.

### Orthology-Based Classification of the Laccase Isozymes From the Literature

As a result of an extensive literature search, information about 37 laccase isozymes from the 12 fungi of the **CorePol_Clade** was collected. The collection included just those laccases for which data on both nucleotide/amino acid sequences and physicochemical/catalytic properties were known.

In case when full or partial nucleotide sequences were available, assignment of laccases to the orthology groups was performed by: (1) the addition to the existed phylogenetic tree via alignment and tree reconstruction (see Synteny-Aware Gene-Tree/Species-Tree Most Parsimonious Reconciliation and Final Laccase Gene-Tree); (2) the reciprocal BLASTn search (Altschul et al., 1990) against collected non-redundant laccase sequences from the fungi of the **CorePol_Clade** (see Synteny-Aware Gene-Tree/Species-Tree Most Parsimonious Reconciliation and Final Laccase Gene-Tree).

In case when just partial amino acid sequences were available, assignment of laccases to the orthology groups was performed by the BLASTp search (Altschul et al., 1990) against collected non-redundant laccase sequences from the fungi of the **CorePol_Clade** (see Synteny-Aware Gene-Tree/Species-Tree Most Parsimonious Reconciliation and Final Laccase Gene-Tree).

All collected information regarding laccase isozymes is placed in the Supplementary Table S2.

### Laccases Recombinant Expression and Purification

Two *T. hirsuta* recombinant laccases, rLacF and rLacD, were obtained in *P. canescens* PCA-10 (niaD^-^) expression system. *P. canescens* transformation and screening was performed according to (Chulkin et al., 2009). Obtained transformants were cultivated for 6 days as described in Abyanova et al. (2010). Culture liquid was separated from mycelium by centrifugation at 10000 *g* for 10 min and used for further purification of enzyme.

The rLacF protein was purified from the culture broth of the ascomycete *P. canescens* by the same methods as previously described for LacA and rLacC in (Savinova et al., 2017), using ion exchange chromatography on DEAE-Toyopearl 650M resin (Tosoh, Japan) eluted with a 5–200 mM gradient of potassium phosphate buffer, pH 6.5, then with a Superdex 75 column (HiLoad 26/600, GE Healthcare, United Kingdom) previously equilibrated with 5 mM potassium-phosphate buffer, pH 6.5. However, after these steps the preparation was not purified from the pigments, therefore, an additional step of purification by hydrophobic chromatography on a Phenyl-Sepharose CL-4B (Sigma-Aldrich, United States) carrier was introduced.

The recombinant rLacD protein was purified from the culture broth of the ascomycete *P. canescens* by the same scheme, but with 20–50 mM Tris-HCl pH 8.0 buffer and without an additional step on a Phenyl-Sepharose.

### Characterization of *T. hirsuta* Recombinant Laccases

The characterization of laccase isozymes including electrophoretic testing, thermal stability, optimal temperature, optimal pH values and substrate specificity assay was performed as described in (Savinova et al., 2017).

Kinetic constants (*K*_M_) for each purified isozyme were determined in 0.1 M citrate-phosphate buffer pH 4.5 at 24°C with various substrates concentrations: 5–2000 μM for ABTS (′ = 436 nm, 𝜀 = 29500 M^-1^⋅cm^-1^) and 2,6-DMP (′ = 470 nm, 𝜀 = 35645 M^-1^⋅cm^-1^), 50–20000 μM for catechol (′ = 410 nm, 𝜀 = 740 M^-1^⋅cm^-1^), 2.5–200 μM for ferulic acid (′ = 314 nm, 𝜀 = 12940 M^-1^⋅cm^-1^) and sinapic acid (′ = 306 nm, 𝜀 = 14640 M^-1^⋅cm^-1^), 125–10000 μM for guaicol (′ = 464 nm, 𝜀 = 6490 M^-1^⋅cm^-1^). Initial rates of product formation in reaction mixtures containing isozymes and the different substrates were measured spectrophotometrically using a PerkinElmer Lambda 35 spectrophotometer (United States). All determinations were carried out in triplicate. Kinetic constants were calculated by non-linear fitting using the Origin-Lab program (Northampton, MA, United States).

## Results and Discussion

### Evolutionary History of the Laccase Genes From the Fungi of the Polyporales Order

To gain insight into the evolutionary history of the laccase genes from the fungi of the Polyporales order, a gene-tree/species-tree reconciliation analysis (Chauve et al., 2013; Nakhleh, 2013; El-Mabrouk and Ouangraoua, 2017) was performed. Essentially, this analysis is comprised of a comparison between two phylogenetic trees – one is the species-tree, depicting the evolutionary history of a group of species, and the other is a gene-tree, depicting the evolutionary history of a particular gene family from these species. By mapping these trees, a reconciliation analysis allows for the recovery of the relative chronological order of major evolutionary events, such as gene losses and duplications that shape the evolution of the gene family.

For the current analysis, 28 wood-decaying fungal species from the Polyporales order were selected. The sample included representative fungi from all four main Polyporales clades (Binder et al., 2013; Justo et al., 2017): the **CorePol_Clade** was represented by 16 fungi; the Antrodia clade was represented by 2 fungi; the Phlebioid clade was represented by 6 fungi, from which 4 fungi formed a distinct Phanerochaecaceae subclade; and the residual Polyporoid clade was represented by 4 fungi. According to the type of decay caused, all fungi were white rot fungi, with the exception of two brown rot fungi from the Antrodia clade (*F. pinicola* and *W. cocos*).

The whole-genome sequences and, consequently, the sequences of all laccase genes were available for all but five of the sampled fungi (*T. ochracea, P. chrysocreas, S. murashkinskyi, A. faginea*, and *A. pallasii*); for these five fungi sequences, the laccase genes were obtained experimentally based on our previous data from targeted laccase pyrosequencing (Moiseenko et al., 2016). In total, there were 153 non-redundant laccase sequences collected, and each fungus except for the representatives of the Phanerochaecaceae subclade contained several non-allelic laccase genes. The use of whole-genomic data together with targeted laccase pyrosequencing data assured that the entire laccase multigene family from each sampled fungus was considered for further laccase gene-tree construction and gene-tree/species-tree reconciliation.

**FIGURE 1:**
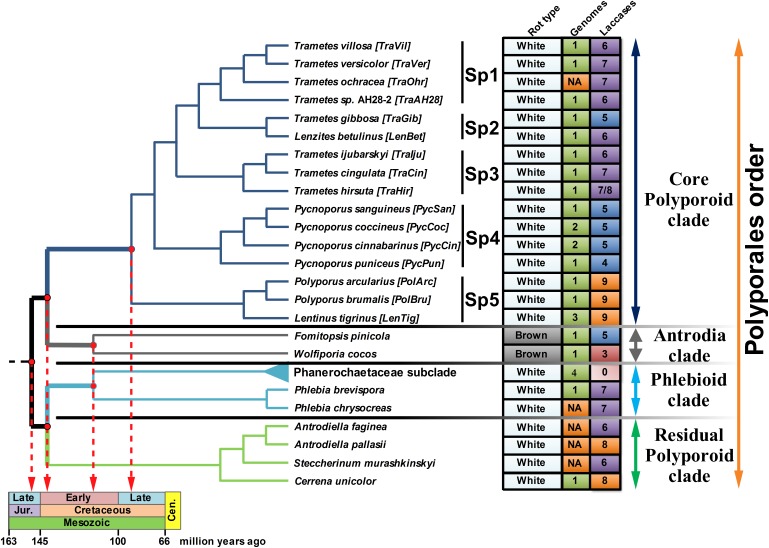
The fungal species-tree. The topology of the tree for the selected fungi of the Polyporales order was extracted from the super-trees in Binder et al. (2013) and Justo et al. (2017). The main clades of the Polyporales order are represented by different colors of the tree branches. The abbreviation is provided for each fungus. Four fungi from the Phanerochaecaceae subclade – *P. carnosa*, *P. chrysosporium*, *P. gigantea*, and *B. adusta*, are collapsed together (the light blue branch). The rot type, number of publicly available genomes (NA - genome was not available) and number of the detected laccase genes are summarized in the right panel; for *T. hirsuta*, 7 functional laccase genes and 1 laccase pseudogene were identified. Tree branches associated with the evolutionary timeline (Garcia-Sandoval et al., 2011; Floudas et al., 2012; Krah et al., 2018) are thickened and projected onto the geological time scale.

The topology of the species-tree for the selected fungi was extracted from the highly reliable super-trees constructed as a part of the PolyPEET project (Binder et al., 2013; Justo et al., 2017) and is depicted along with the relevant data regarding these fungi in Figure 1. For the upcoming discussion, the **CorePol_Clade** on the species-tree was subdivided into five distinct subclades – **Sp 1–5.**

For the inference of the laccase gene-tree, a two-stage procedure was adopted. The initial gene-tree was constructed by the maximum likelihood method, based solely upon the nucleotide alignment of the laccase sequences, and the levels of support for each branch on the tree were assessed by bootstrapping. The final laccase gene-tree was obtained as a result of the synteny-aware gene-tree/species-tree most parsimonious reconciliation analysis with simultaneous local rearrangements of the low supported branches (Lafond et al., 2013a,b; Wu et al., 2013; Noutahi et al., 2016). In general, the low supported branches were rearranged in a manner that: (1) minimized the number of gene loss/duplication events; and (2) placed the most recently duplicated and, hence, the most closely located of the chromosome genes in the closest topological proximity on the gene-tree.

**FIGURE 2:**
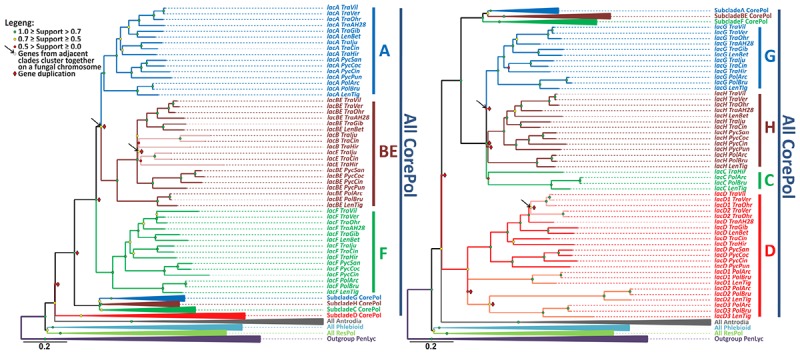
The laccase gene-tree. The phylogenetic relationships of the laccase genes from the selected fungi of the Polyporales order inferred under the maximum likelihood criterion with a subsequent rearrangement of low supported branches by the synteny-aware gene-tree/species-tree most parsimonious reconciliation procedure. Bootstrap values at each node are color-coded, and indicators of the inferred gene duplications and synteny are placed near the corresponding nodes (see the legend). The clades that contains all laccase genes from the fungi of the core Polyporoid clade (**All CorePol**) is serially expanded (subclades **A**-**H**). Expansion of the collapsed clades **All Antrodia**, **All Phlebioid,** and **All ResPol** contains all of the laccase genes from the fungi of the Antrodia, Phlebioid, and Residual Polyporoid clades, respectively, and can be found in Supplementary Figure S1, along with the expanded **Outgroup PenLyc** clade containing the three laccase genes from *P. lycii* that were used as an outgroup. For the fungal species abbreviations, refer to Figure 1.

**FIGURE 3:**
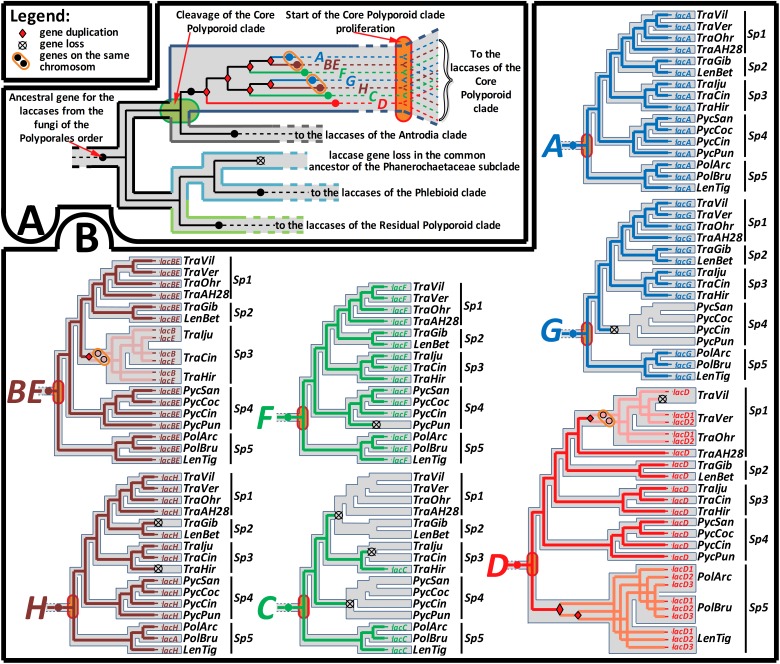
Summary of the gene-tree/species-tree reconciliation analysis. The relative sequence of events in the evolution of the laccase gene family from the fungi of the Polyporales order, recovered by the synteny-aware gene-tree/species-tree most parsimonious reconciliation procedure. For the symbols that marked gene loss and duplication events and location of the duplicated genes on the same chromosome, refer to the figure legend. **(A)** Represents the early sequence of evolutionary events from the emergence of the Polyporales order until its splitting into four main clades. Special emphasis is placed on the detected multiple gene duplications in the core Polyporoid clade, which occurred between its cleavage and start of proliferation. **(B)** Represents the detailed sequence of evolutionary events from the start of the core Polyporoid clade proliferation until the present. The evolutionary history of each duplicated copy **(A–H)** of the ancestral gene is separately mapped onto the relevant part of the species-tree (see Figure 1). Simultaneous mapping of all seven genes can be found in the Supplementary Figure S2. For the fungal species abbreviations, refer to Figure 1.

The relevant part of the final laccase gene-tree is represented in Figure 2, and the remainder of the tree is shown in the Supplementary Figure S1. The results of the gene-tree/species-tree reconciliation analysis are summarized in Figure 3, and for the entire analysis, see Supplementary Figure S2.

The performed gene-tree/species-tree reconciliation analysis proposed that all laccases in the fungi of the Polyporales order were derived from a single ancestral gene, and this gene did not duplicate until the splitting of this order into its four main clades (Figure 3A and Supplementary Figure S2). This absence of the early ancestral gene duplications is manifested by the arrangement of the laccases from the fungi of the different main clades into their distinct subclades (**All CorePol**, **All Antrodia**, **All Phlebioid,** and **All ResPol)** on the gene-tree (Figure 2 and Supplementary Figure S1).

The reconciliation analysis also suggests that, after the splitting of the four main Polyporales clades was completed, the single laccase gene that came into the **CorePol_Clade** duplicated six times before the clade started to proliferate. As a result of such multiple early duplications, all of the laccase genes from the fungi of the **CorePol_Clade** formed seven distinct subclades (**A-H**) (Figure 2), and phylogenetic relationships of these genes within the subclades resembled phylogenetic relationships between the corresponding fungal species (Figure 1).

In contrast to the **CorePol_Clade**, the single laccase gene that came into the Phlebioid clade did not duplicate until the splitting of the Phanerochetacea subclade. Such sequence of events provides a clear explanation of the absence of laccase genes in the fungi of this subclade, due to the single gene loss in their most recent common ancestor (Figure 3A and Supplementary Figure S2). Indeed, the possibility of almost simultaneous (in a geological time scale) multiple gene losses in all representatives of this subclade are highly unlikely. Moreover, no evidence of the recent laccase gene losses, such as laccase pseudogenes and gene fragments, could be detected in the genomes of these fungi.

Unfortunately, a limited number of the closely related lineages for the fungi from the residual Polyporoid and Antrodia clades did not allow for the identification of the relative time spans for the duplications of their ancestral laccase genes. Nevertheless, in the case of the Antrodia clade, since laccases from the different fungi of this clade formed distinct subclades on our gene-tree (Supplementary Figure S1), it can be hypothesized that a single laccase gene that came into this clade began to duplicate in the distinct lineages formed after the beginning of the clade proliferation.

In the case of the **CorePol_Clade**, a denser species sampling allowed for the investigation of the most recent evolution of its laccase genes. A detailed analysis of the subclades formed by the laccase genes from these fungi (Figure 2 and Supplementary Figure S2) revealed that the beginning of these clade proliferation losses and duplications of laccase genes were relatively uncommon events: in subclade **A**, neither gene losses nor duplications were detected; in subclade **BE**, one gene duplication in the common ancestor of the fungi from **Sp 3** was detected; in subclade **F**, one gene loss in the lineage going to *P. puniceus* was detected; in subclade **G**, one gene loss in the common ancestor of the fungi from **Sp 4** was detected; and in subclade **H**, two gene losses in the lineages going to *T. hirsuta* and *T. gibbosa* were detected. Interestingly, in the genome of *T. hirsuta*, the lost gene, *lacH*, is still readily detectable in the form of a pseudogene (Moiseenko et al., 2018).

Two exceptional laccase subclades that deserve special attention are subclades **C** and **D**. In subclade **C**, three parallel early gene losses were detected: first, in the common ancestor of the fungi from **Sp 1** and **Sp 2**; second, in the common ancestor of *T. ljubarskyi* and *T. cingulate*; and third, in the common ancestor of the fungi from **Sp 4**. Given the loss of the *lacH* gene in *T. hirsuta*, it can be speculated that the retention of *lacC* by this fungus may be a compensatory event.

In subclade **D,** one gene loss occurred in the lineage going to *T. ljubarskyi*; one gene duplication occurred in the common ancestor of the three fungi from **Sp 1** (without *Trametes* sp. AH28-2), and one copy of this duplicated gene was lost in the lineage going to *T. villosa*; and two subsequent gene duplications occurred in the common ancestor of **Sp 5**. Additionally, it should be mentioned that during phylogenetic analysis, there was equally supported positioning of subclade **D** at the same node as the laccases from the brown rot fungi. However, such positioning demanded one extra gene loss in the common ancestor of all fungi of the core Polyporoid and Antrodia clades and, therefore, was less parsimonious than the presented one.

In putting the evolutionary trend over the previously inferred (Garcia-Sandoval et al., 2011; Floudas et al., 2012; Krah et al., 2018) historical timeline of the Polyporales order (Figure 1), it is evident that, from the emergence of this order near the end of the Jurassic until its splitting into four main clades near the early Cretaceous, only one copy of the laccase gene was present in the genomes of the ancestral fungal population. Duplications of this single-copy ancestral gene can be approximately assigned to the second half of the early Cretaceous, the time by which the angiosperm plants were undergoing a major radiation that would eventually lead to their predominance over the gymnosperms at the end of the Cretaceous (Lupia et al., 1999; Coiffard et al., 2012). Although angiosperms provided a new mega-niche for the wood-rot fungi, especially white rot fungi (Krah et al., 2018), they also presented a new challenge. In contrast to the gymnosperms (softwood) that generally deposit lignin primarily derived from guaiacyl monomers (G-type units) with a small amounts of hydroxyphenyl (H-type) units, the lignin of the angiosperms (hardwood) are composed of guaiacyls and syringyls (S-type units) in approximately equal ratios (Sarkar et al., 2009; Weng and Chapple, 2010; Espiñeira et al., 2011; Popper et al., 2011; Wang et al., 2013). This new type of lignin was more resistant to degradation (Skyba et al., 2013) and resulted in a greater variety of degraded phenolic derivatives that needed to be detoxified.

It can be hypothesized that the multiple duplications of the laccase genes in the Polyporales near the second half of the early Cretaceous were related to the undergoing proliferation of the angiosperm plants. Although by that time, wood-rot fungi already had several copies of lignin-degrading peroxidase genes (Floudas et al., 2012; Ruiz-Dueñas et al., 2013), this was not sufficient for the rapid, effective, and competitive exploration of the newly formed ecological niche. Moreover, previous studies suggest that during the time span under discussion, there was a comparatively smaller number of duplications of peroxidase genes; extensive parallel duplication of which, in the different lineages of the Polyporales order, can be attributed to the later time periods (Floudas et al., 2012; Ruiz-Dueñas et al., 2013). It is also probable that the partial oxygen pressure that was gradually increasing from the beginning of the late Cretaceous until its drastic drop at the beginning of the Tertiary (Gale et al., 2001; Hay and Floegel, 2012) substantially spurned proliferation of peroxidases in favor of laccases. The aerobic atmosphere of the planet has caused surface iron to be converted into oxyhydroxide polymers of very sparing solubility (Janusz et al., 2013). Consequently, wood-rot fungi were forced to prefer “Cu-dependent” laccases over the “Fe-dependent” peroxidases during that time span.

Hence, for wood-rot fungi at the early stages of adaptation to the angiosperm-plant environmental dominance, several copies of laccase genes were presumably more selectively advantageous than the expansion of the peroxidase multigene family. In the case of the fungi from the Phanerochaecaceae subclade, which lost their ancestral laccase gene early in their evolution, it can be speculated that the expansion of structurally similar and evolutionary-related multigene families of MCO in this fungi could compensate for the loss of the *sensu stricto* laccases (Martinez et al., 2004), although additional analysis on the time of duplications for these MCO genes is necessary for more definite conclusions.

### Orthology-Based Classification of the Laccase Isozymes From the Fungi of the Core Polyporoid Clade

For the fungi of the **CorePol_Clade**, the level of detail achieved for the laccase gene-tree allows us to use this tree as a base for the classification of laccases from these fungi into OG. Since a relatively small number of lineage-specific duplications of laccase genes was detected, clades **A–H** can be generally seen as a collection of very closely related orthologs. Consequently, the constructed phylogenetic tree can be seen as a scaffold for positioning laccase isozymes within the previously described properties into the well-defined OG **A–H**, which include laccases from clades **A–H** on the gene-tree.

**FIGURE 4:**
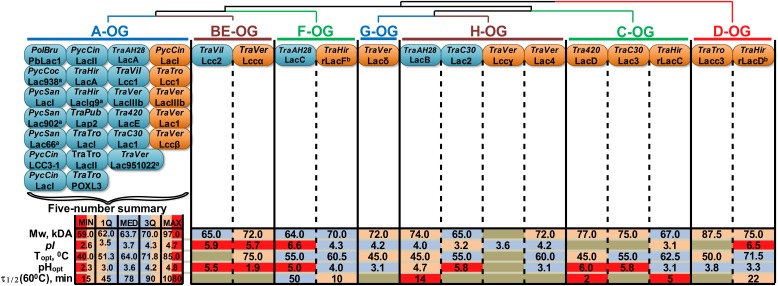
Orthology-based classification of laccase isozymes with the summary of their physicochemical properties. The phylogenetic relationships between different OG are represented by the dendrogram. The blue and orange oval shapes represent different laccases of native or recombinant isozymes (respectively); the proteins nomenclature provided in accordance with the source (see Supplementary Table S2), unless otherwise is indicated; ^a^named according to the strain number; ^b^data obtained in this study. TraPub, TraTro, Tra420, TraC30 designate *Trametes pubescens*, *Trametes trogii*, *Trametes* sp. AH28-2 and *Trametes* sp. C30, respectively. For other abbreviations, refer to Figure 1. For group **A**, a five-number summary is provided, with MIN, MAX, MED, 1Q and 3Q being the minimum, maximum, median, first and third quartiles, respectively. The data regarding laccases from groups **BE**-**H** are color-coded according to their locations in the corresponding five-number summary intervals.

As a result of an extensive literature search, 37 laccase isozymes from 12 fungi of the **CorePol_Clade** were assigned to the abovementioned OG (Figure 4 and Supplementary Figure S2).

It is obvious that almost all native laccase proteins that were described in the literature until now belong to group **A**. These laccases are natively produced by the fungi of the **CorePol_Clade** under great varieties of growth conditions, and their transcriptional level is relatively stable during all cultivation stages (Moiseenko et al., 2018). In contrast, the native production of laccases from other groups was always observed under very specific inductive conditions such as induction by 2,5-xylidine for group **BE** laccases from *T. villosa* (Yaver et al., 1996); cocultivation with *Trichoderma* sp. ZH1 for group **F** laccases from *Trametes* sp. AH28-2 (Zhang et al., 2006); combined induction by copper and *p*-hydroxybenzoate for group **H** laccases from *Trametes* sp. C30 (Klonowska et al., 2002); and growth on cellobiose-based medium containing 3,5-dihydroxytoluene for group **H** laccases from *Trametes* sp. AH28-2 (Xiao et al., 2004). Moreover, the transcription of laccases from groups **BE-H** was shown to be rather mosaic and characterized by the sharp increasing and decreasing of expression levels, depending on both growth conditions and cultivation stages (Moiseenko et al., 2018).

Unfortunately, orthology-based classifications of laccase isozymes revealed a substantial gap in the current knowledge about their physicochemical and catalytic properties. A small number of purified laccase isozymes from groups **BE-H** and the different conditions (e.g., substrates, buffers, pH and temperature) under which the properties of these laccases were measured do not allow for the inference of any definite trends. This situation is especially prominent in the case of catalytic properties, which were completely impossible to systematize, since the catalytic constant values are very sensitive to the measurement conditions, and there were almost no studies conducted under the exact same conditions. Nevertheless, by comparison to the statistical ranges of the laccases from group **A**, several observations about the physicochemical properties of laccase isozymes from other groups can be highlighted: all of the currently described laccases of groups **BE** and **F** were characterized by unusually high p*I* values – near six; the pH_opt_ values of many laccases from groups **BE-H** were determined in neutral rather than acidic regions; and laccases from groups **BE-H** were almost always characterized with lower thermostability.

In addition to being capable of bringing order to the very scattered data regarding different laccase isozymes from different fungi, the systematization of laccases based on their orthologous relationships potentially has predictive value in the view of so-called “orthology conjecture”. Being one of the most significant insights from the modern development of phylogenomics, “orthology conjecture” implies that orthologous genes are functionally more similar than paralogous ones (Altenhoff et al., 2012; Chen and Zhang, 2012; Gabaldón and Koonin, 2013). Hence, the acceptance of the orthology conjecture potentially allows for the extrapolation of experimentally obtained information about the properties of laccase isozymes in one fungus onto their OG representatives in the related fungi, at least within the **CorePol_Clade**. Nevertheless, as suggested by our data analysis, to gain full benefit from the orthology conjecture, the systematic obtaining and characterization of laccase isozymes from groups **BE-H** are vital.

### Physicochemical and Catalytic Properties of Four Laccase Isozymes From *T. hirsuta*

The existence of an excellently resolved species tree, with a presumably small number of species-specific duplications of laccase genes and high biotechnological potential, makes the fungi of the Polyporales order an attractive target for in-depth study of laccase multigene family diversification. Denser sampling of the **CorePol_Clade** also allows for the tracing of volatility characteristics related to laccase biological specialization. We hypothesize that the obtained information regarding the properties of laccase isozymes can be extrapolated to their orthologous clade representatives, at least within the **CorePol_Clade** (Figure 1). Therefore, the data systematization of properties and structure of laccases within multigene families is necessary.

There is not much data that exist on different laccase isozymes properties. The maximum number of laccase isozymes from the fungi of *Trametes* genus was obtained and characterized for *T. versicolor* 3086, namely, four laccases from different OG (**A, BE, H,** and **G**) (Supplementary Table S2) (Necochea et al., 2005; Koschorreck et al., 2008). Here, we present data regarding physicochemical properties of four laccase isozymes from *T. hirsutà* 072 belonging to three other OGs: recombinant isozymes belonging to the **F-**OG (rLacF) and **D-**OG (rLacD**)** were obtained during this study, while recombinant isozymes belonging to the **C-**OG (rLacC) and native isozymes belonging to the **A-**OG (LacA) were obtained and partially characterized previously (Savinova et al., 2017). Additionally, we compare the catalytic properties of four *T. hirsuta* 072 laccase isozymes, which allowed us to partially bridge the detected gap in the current knowledge about laccases from groups other than **A**-OG.

A comparative analysis of *T. hirsuta* laccase isozymes (Supplementary Table S2) showed the differences in their specific characteristics. The optimal pH range for the oxidation of phenolic substrates for **rLacC** and **rLacF** was shifted to a less acidic region (5.0–5.25 and 4.25–5.0, respectively) as compared to **LacA** and **rLacD** (3.75–4.0 and 3.75–4.5, respectively). When using the non-phenolic ABTS substrate optimal pH values of **rLacF**, which was also less acidic then for other isozymes (2.5–3.5 against 2.25–2.75 respectively), and for which pH optima were common in most fungal laccases (Baldrian, 2006). The **rLacD** isozyme of *T. hirsuta* was also distinguished in pI value, which was strongly shifted to the neutral pH range (6.5) compared to the other isozymes. The **rLacF** had a less acidic pI (4.3) than laccases **A** and **C**.

The analysis of the kinetic constants (*K*_M_) for 6 typical laccase substrates: ABTS, catechol, 2,6-dimethoxyphenol (DMP), ferulic acid, synapic acid and guaiacol was conducted (Table 1).

**Table 1:** Kinetic constants (*K*_M_) of the laccase isozymes from *T. hirsuta* 072.

Substrate	λ, (nm)	𝜀, (M^-1^⋅cm^-1^)	*K*_M,_ μM			
			LacA^∗^	rLacC	rLacD	rLacF
ABTS	436	29500	17 ± 2	534 ± 33	37 ± 2	88.5 ± 6
Catechol	410	740	183 ± 16	16299 ± 1719	280 ± 21	3627 ± 566
*S-type phenolic compounds:*						
2,6-DMP	470	35645	24 ± 2	589 ± 75	17 ± 1.3	79 ± 11
Synapic acid	306	14640	17 ± 2	71 ± 14	5 ± 0.6	33 ± 9
*G-type phenolic compounds:*						
Ferulic acid	314	12940	28 ± 3	173 ± 38	78 ± 13	118 ± 16
Guaiacol	464	6490	173 ± 15	15742 ± 337	931 ± 64	2766 ± 368


*^∗^Glazunova et al. (2018b).*

Among the substrates investigated, all isozymes showed the lowest affinity toward guaiacol and catechol (the highest *K*_M_ values) and the highest affinity toward synapic acid. It is interesting that for the minor **rLacD**, the affinities toward 2,6-DMP and synapic acid (S-type substrates) were slightly higher than for the major **LacA** isozyme, which is a characteristic feature of the *Steccherinaceae* group laccases (Glazunova et al., 2018a). It also should be noted that among the minor laccases (**rLacC, rLacF,** and **rLacD**), both S-type substrates were better for **rLacD** and **rLacF**, while **rLacC** affinity toward 2,6-DMP was significantly poorer than it was toward synapic acid.

The shown differences in *K*_M_ values of different laccases may be one more example of the evidence for multiple laccase gene duplications as a mechanism of evolutionary adaptation of the fungus to changing environmental conditions. The proliferation of new plant groups provided new challenges in lignin degradation, as well as to the detoxification of a broad spectra of degraded phenolic derivatives, thereby confirming the inferences described here earlier (see Evolutionary History of the Laccase Genes From the Fungi of the Polyporales Order). The *T. hirsuta* is a fungal cosmopolite capable of growing on different types of wood and plant substrates during different stages of lignin decomposition, it possess a successful adaptation strategy, probably also through the use of different laccase isozymes with different substrate specificities.

It should also be noted that the ratio of monomeric units of lignin may not only depend on the plant origin but also vary in different plant growth conditions, plant age and even among cell types on the same plant (Moura et al., 2010; Wang et al., 2013; Munk et al., 2015). Comparison of the results of a substrate specificity test of *T. hirsuta* 072 laccase isozymes (Table 2) confirms the correlation of differences in the properties of isozymes to the phylogenetic proximity of the laccase genes. Thus, the products of the *lacA* and *lacF* genes, which refer to the same evolutionary group, show the most similar substrate specificity and, apparently, can perform similar roles in the fungus. The *lacC* gene product has the narrowest substrate oxidation range and is not intended for the oxidation of substrates containing amino groups (Savinova et al., 2017). Previously, we had shown that the expression of both *lacC* and *lacF* genes in *T. hirsuta* can be induced by different products of lignin degradation and suggested helping the function of these laccases for the mainly produced LacA (Moiseenko et al., 2018). Given the current results, we can speculate that the **rLacC** and **rLacF** most likely are accessories to LacA. Being expressed under the increased concentration of phenolic moieties in the fungal environment, according to *K*_M_ values, both **rLacC** and **rLacF** begin to fully operate at higher (compared to LacA) concentrations of phenolic compounds.

**Table 2:** Substrate specificity test for laccase isozymes from *T. hirsuta* 072.

Substrates	Types of lignin monomers	*T. hirsuta* 072 Isozymes			
		LacA^∗∗^	rLacC^∗∗^	rLacD	rLacF
Gallic acid	S-type	++	++	++	++
Syringic acid		++	++	++	++
Ferulic acid	G-type	++	++	+	++
Vanillic acid		++	+	+/-	+
Veratryl alcohol		–	–	–	–
Vanillin		++	+/-	+	++
Guaiacol		++	++	++	++
p-Coumaric acid	H-type	++	++	–	++
Orcinol	–	++	+/-	+/-	+
2,5-Xylidine		++	+/-	++	+
*o*-Toluidine		++	+/-	+	+


*The designations are as follows: “++”, indicates explicit changes in the spectra relative to the control; “+”, visible changes with respect to the control; “+/-“, moderate visible changes; “-“, no visible spectra changes; ^∗∗^Savinova et al. (2017).*

Based on the data obtained, an interesting feature of **rLacD** was also highlighted. **rLacD** was unable to modify *p*-coumaric acid and has a poorer ability to modify ferulic and vanillic acids in comparison with the other isozymes. Previously, we had shown that the expression of the *lacD* gene can be induced by nutritional stress, which in turn can cause oxidative stress (Moiseenko et al., 2018). In view of the presented data, it can be speculated that the *K*_M_ of **rLacD** was evolutionary optimized for stress conditions. Being very rapidly activated due to its high substrate affinities (in the case of ferulic acid), this laccase probably performs very slow free-radical formation, which does not cause additional stress but ameliorates the already present stress by promoting free radical copolymerization.

It has been suggested that the variability of enzyme efficiency is dependent not only on the enzyme’s function within the organism but also on the specific metabolic context (Bar-Even, 2012). Although efficiency is considered to be the main parameter that is optimized during the evolutionary process, in a number of cases, it has been shown that *K*_M_ can be optimized (selected according to specific values) in order to coincide with the expected concentration of the substrates (Albery and Knowles, 1976; Aharoni et al., 2005). Moreover, it appears that for many enzymes, significant reductions in rate have no effect, and only relatively large reductions in catalytic efficiency hinder organismal fitness (Bar-Even, 2012). Although promiscuous activities are often orders of magnitude lower than the native activity, they may provide a selective advantage (Aharoni et al., 2005; James and Tawfik, 2008).

On the other hand, a decrease (or total absence) in the oxidizing ability toward G- and H-types lignin derivatives (in particular ferulic acid and *p*-coumaric acid) is characteristic of the ascomycetes laccase from *M. thermophila*, as well as of plant and bacterial laccases (Reiss et al., 2013). Thus **rLacD**, likely, can perform similar functions as the functions of bacterial or plant laccases.

Using the example of four *T. hirsuta* 072 laccases and the laccases of other fungi of the genus *Trametes* (**CorePol_Clade**s), we aimed to identify the main features of individual clades, as well as understand how the phylogenetic similarity corresponds to the biochemical similarity in the multigenic family, namely, whether these distributions over clades are preserved with respect to the properties of isozymes. Comparison of the properties of laccases from different clades made it possible to reveal some patterns (Supplementary Table S2). In general, laccases from clades A, BE, and F had a lower molecular weight than laccases from clades C, G, and H. Laccases from clade D had the largest molecular weight (≈75–90 kDa). Since the predicted molecular weights of proteins differed insignificantly, it can be assumed that such variations are associated with the various glycosylation of enzymes. Unlike the other laccases, the laccases from clades A and BE are characterized by the simultaneous production of multiple isoforms, which are distinguished by glycosylation, since it is precisely due to these laccases that there is a presence of multiple bands on gels, even during recombinant production.

The analysis of the kinetic constants showed that the *K*_M_ values for the ABTS substrate generally correspond with the phylogenetic relationship (gene tree): laccases from clades A, BE and F are characterized by a greater affinity for this substrate than laccases from clades C, G, and H. For phenolic substrates (2,6-DMP, Guaiacol, and Catechol), this is not true. However, a relatively small amount of information about these substrates for different laccases should be considered. Nevertheless, the two characterized laccases from clade F (*T. hirsuta 072* and *Trametes* sp. AH28-2) show a high affinity for the 2,6-DMP substrate (similar to laccase A).

In terms of catalytic properties, clade D shows a great heterogeneity for its characterized representatives. Based on the data for rLacD *T. hirsuta* 072, we hypothesized that the main substrates of these isozymes may be S-type lignin monomers. Considering the previous results on the expression of the *lacD* gene (Moiseenko et al., 2018), the isolated location of clade D on the phylogenetic tree and the specific biochemical properties of rLacD of *T. hirsuta* that are not common for fungal *sensu stricto* laccases, it can be assumed that the laccases of this orthology group significantly differ in the functions performed in the fungi. However, in order to reveal patterns of substrate preferences, the laccase of this clade needs a more detailed description of a wider range of its representatives.

The protein alteration toward a new function involves the transitions from a specialized enzyme into a generalized intermediate and, ultimately, a new, ‘respecialized’ enzyme (Matsumura and Ellington, 2001; Aharoni et al., 2005). Under changing environmental conditions, additional activity in an existing protein provides the organism with a selective advantage leading to its survival and further development (Jensen, 1976). The existing hypothesis presupposes the following mechanism of protein divergence: an initial beneficial mutation, rendering it as generalized by increasing the protein’s promiscuous activity to a level sufficient for survival, while maintaining the original activity largely intact, further gene duplication, and then the divergence of a new gene with respect to sequence and function (Lynch and Katju, 2004). Thus, gene duplication is the driving force of existing protein evolution, and duplicated genes can serve as a starting point for the diversification to a new function (James and Tawfik, 2008). However, for the emergence of a new enzyme function, it is necessary that the protein exhibits some new, uncharacteristic activity. In the case of laccases, changes in their biochemical properties and substrate preferences likely indicate changes in the primary function of the ancestral enzyme driven by the need of adaptation to the new and rapidly changing environmental conditions.

In the course of this study, there were two basic tasks to solve: on the one hand, we tried to find out the driving force of the evolutionary processes (genes duplication, divergence, loss, etc.) occurring within the multigene laccase family of the Polyporales genus, reveal the proliferation basis of this family; on the other hand, we intended to highlight the motives of laccases diversification through the comparison of the isoenzymes properties. Striking differences in physical-chemical properties of enzymes within one fungal species led us to a suggestion that the start of Polyporoid clade laccases proliferation and formation of distinct OGs containing laccases with distinct properties is a sign of functional variability of isoenzymes. Our analysis of the phylogenetic relationships within the family confirms the idea that the duplication of genes and diversification of laccases functions were parallel interrelated processes. It should be noted that for the scientifically sound generalization of our conclusions on the laccases of the same orthology groups from other fungi, additional investigations are necessary. We propose that our systematic orthology-based approach to the study of different laccase isozymes can be a guideline for such investigations.

## Conclusion

The evolutionary analysis of the *sensu stricto* laccase genes of Polyporales conducted in this work suggests that all Polyporales laccases derived from a single ancestral gene. Extensive duplications of this gene began almost immediately after the splitting of the Polyporales order into its four main clades, and continued with the evolution of the angiosperms, which may be a consequence of the conquest of new ecological niches by the fungi. The fungi of the **CorePol_Clade** achieved a level of detail in the laccase gene-tree that allows the use of this tree as a base for the classification of laccases into seven OGs, proposing multigene family diversification connected to environment changes leading to the adaptation of existing enzymes toward new life conditions.

## Author Contributions

DV, TT, and TF conceived the study. KM, DV, and AC provided the methodology. OS, KM, AC, and EV contributed to the investigation process. DV, KM, and OS wrote the original draft of the manuscript. DV, KM, OS, TT, and TF wrote, reviewed, and edited the manuscript. TT and TF supervised the work. All authors read and approved the final manuscript.

## Conflict of Interest Statement

The authors declare that the research was conducted in the absence of any commercial or financial relationships that could be construed as a potential conflict of interest.

## Footnotes

1 https://wordpress.clarku.edu/polypeet/

2 https://www.phylo.org/

## Supplementary Material

FIGURE S1 The remainder of the laccase gene-tree. The following abbreviations are used: FomPin, F. pinicola; WolCoc, W. cocos; PhlChr, P. chrysocreas; PhlBre, P. brevispora; AntFag, A. faginea; AntPal, A. pallasii; SteMur, St. murashkinskyi; CerUni, C. unicolor; PenLyc, P. lycii.

FIGURE S2 The gene-tree/species-tree reconciliation analysis. For the fungal species abbreviations refer to Figure 1.

TABLE S1 The laccase sequences used in this study.

TABLE S2 Properties of various laccase isozymes from different source.

## Abbreviations

2,6-DMP: 2,6-dimethoxyphenol
ABTS: 2,2’-azino-bis(3-ethylbenzothiazoline-6-sulfonic acid) diammonium salt
CorePol_Clade: Core Polyporoid clade
G-type: Guaiacyl-type
H-type: p-Hidroxyphenyl-type
ITS: internal transcribed spacer
MCO: multicopper oxidases
OG: orthologous group
Sp 1-5: Core Polyporoid subclades
S-type: Syringyl-type substrates
