# Supplementary figures and images for: Evolutionary Relationships Between the Laccase Genes of Polyporales: Orthology-Based Classification of Laccase Isozymes and Functional Insight From Trametes hirsuta
Source: Front Microbiol. 2019 Feb 6;10:152. doi: 10.3389/fmicb.2019.00152 (PMC6374638; doi:10.3389/fmicb.2019.00152)

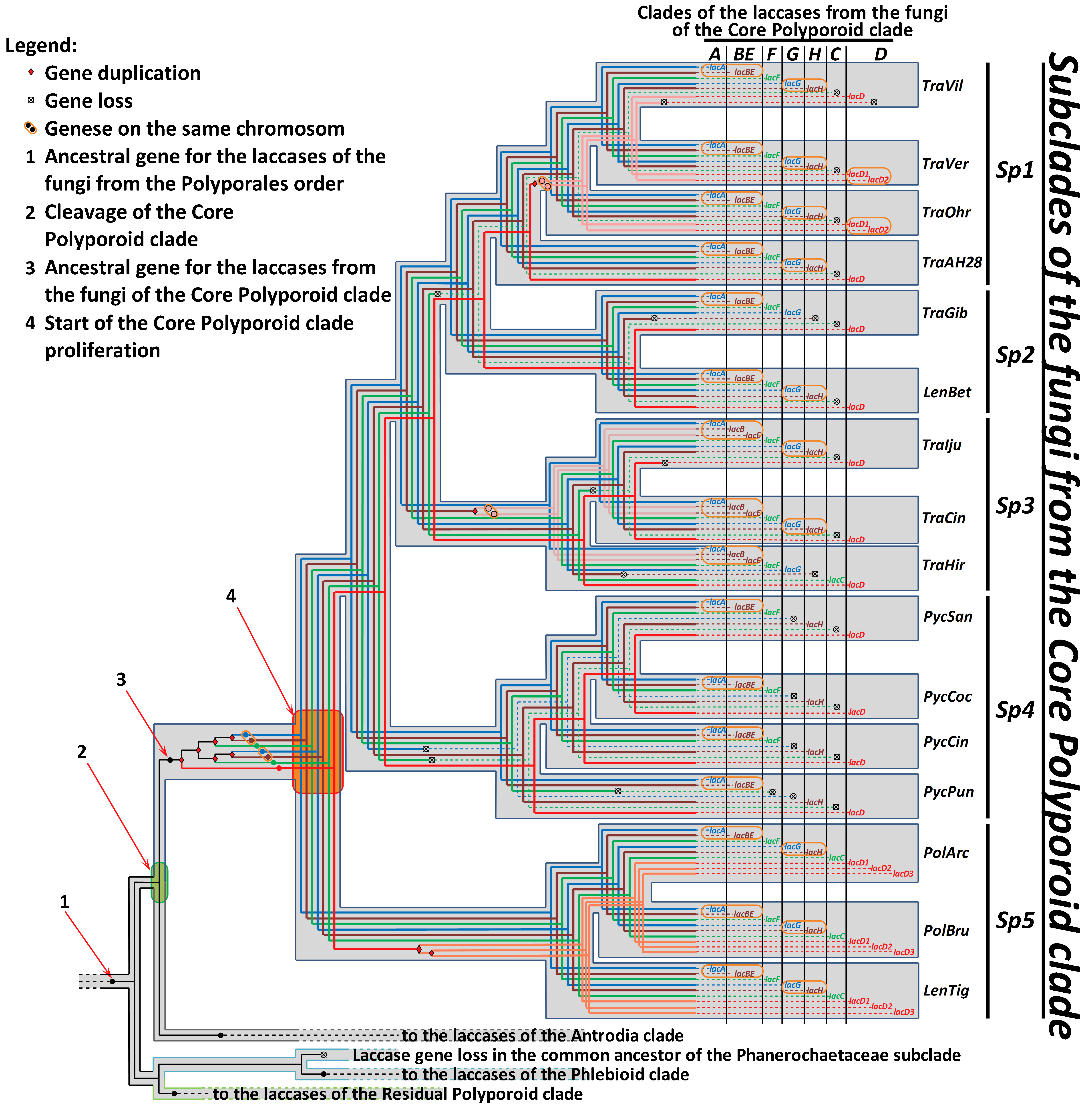

Supplement: FIGURE S2 — The gene-tree/species-tree reconciliation analysis. For the fungal species abbreviations refer to Figure 1. [file Image_2.TIF]
