# Supplementary material for: Evolutionary Relationships Between the Laccase Genes of Polyporales: Orthology-Based Classification of Laccase Isozymes and Functional Insight From Trametes hirsuta
Source: Front Microbiol. 2019 Feb 6;10:152. doi: 10.3389/fmicb.2019.00152 (PMC6374638; doi:10.3389/fmicb.2019.00152)
Supplement: TABLE S2 — Properties of various laccase isozymes from different source. [file Table_2.DOCX]

**Table S2.** Properties of various laccase isozymes from different sources.

# The designations are as follows: ^a^-ABTS, ^b^-catechol, ^c^-2,6-dimethoxyphenol, ^d^- guaicol, ^e^-*o*-tolidine, ^f^- syringaldehyde, * - possible isoforms of one gene product, n/a - no sequence; Not A clade, native; A clade, native; Not A clade, recombinant; A clade, recombinant

| **Strain** | **Protein** | **Origin** | **Clade** | **Мw, kDa** | ***pI*** | **Topt, °C** | **pHopt** | **Thermostability (τ_1/2_), min** | **К_м_, µМ** | **Ref.** |
| --- | --- | --- | --- | --- | --- | --- | --- | --- | --- | --- |
| *Pycnoporus (Trametes) sanguineus* CS43 | LacI | N | A* | 68 | 4.7 | 70^a^ | 2.5^a^; 3.5^c^; 4.0^d^ | 1080^a^ (60 °С) | 6.9^a^; 89.2^c^; 1484.5^d^ | (Ramírez-Cavazos et al., 2014) |
|  | LacII | N | A* | 66 | 4.6 | 60^a^ | 2.0^a;^ 3.0^c^; 4.0^d^ | 135^a^ (60 °С) | 12.2^a^; 191.6^c^; 1100.8^d^ |  |
| *Pycnoporus sanguineus CeIBMD001* | Lcc | N | n/a | 68 | 7.0-7.08 | - | - | 137^a^ (60 °С) | 238.55^a^; 693.08^d^ | (Dantán-González et al., 2008) |
| *Trametes sanguinea M85-2* | - | N | n/a | 62 | 3.5 | 60^c^ | 5.0^c^ | - | - | (Shinagawa, 1995) |
| *Pycnoporus coccineus IFO-4923* | Lcc | N | n/a | 70 | 3.5 | - | - | - | - | (Oda et al., 1991) |
| *Pycnoporus coccineus BRFM 938* | Lac938 | N | A | 61.8 | - | 65^a^ | 4.5-5.0^a^; | 200^a^ (65 °С) | 26^a^ | (Uzan et al., 2010) |
| *Pycnoporus sanguineus BRFM 902* | Lac902 | N | A | 62.9 | - | 65^a^ | 4.5-5.0^a^; | 160^a^ (65 °С) | 32^a^ |  |
| *Pycnoporus sanguineus BRFM 66* | Lac66 | N | A | 59.5 | - | 71^a^ | 4.5^a^ | 200^a^ (65 °С) | 33^a^ |  |
| *Polyporus brumalis ibrc05015* | PbLac1 | N | A | 63.4 | - | 40^a^ | 4.0^a^ | - | 20^a^; 70^c^; 10700^d^ | (Nakade et al., 2010; Ryu et al., 2008) |
| *Polyporus brumalis KFRI20912* | - | N | n/a | 70 | - | 20^e^ | 4.0^e^ | - | 685^e^ | (Kim et al., 2012) |
| *Trametes (Pycnoporus) cinnabarina* ATCC 200478 | LCC3-1 | N | A | 76.5 | 3.7 | - | 4.0^d^ | 60^d^ (70 °С) | - | (Eggert et al., 1996; Temp et al., 1999) |
| **Strain** | **Protein** | **Origin** | **Clade** | **Мw, kDa** | ***pI*** | **Topt, °C** | **pHopt** | **Thermostability (τ_1/2_), min** | **К_м_, µМ** | **Ref.** |
| *Trametes sp.* AH28-2 | LacA | N | А | 62 | 4.2 | 50^d^ | 4.5^d^ | 27^d^ (75 °С) | 25^a^; 25,5^c^; 420^d^ | (Xiao et al., 2003) |
|  | LacB | N | H | 74 | 4.0 | 45^d^ | 4.7^d^ | 14^d^ (60 °С) | 177^a^; 109,8^c^; 1249^d^ | (Xiao et al., 2004) |
|  | LacC | N | F | 64 | 6.6 | 55^d^ | 5.0^d^ | 50^d^ (60 °С) | 50^a^; 56^c^; 1138^d^ | (Zhang et al., 2006) |
| *Pycnoporus cinnabarinus strain ss3.* | Lac I | N | A | 70 | 3.7 | - | 4.0^f^ | - | 55^a^ | (Otterbein et al., 2000) |
|  | Lac I | R | A | 70 | 3.7 | 63^a^ | 2.5^a^; 4.0^f^ | 100^a^ (65 °С) | 55^a^ | (Record et al., 2002) |
|  | Lac II | N | A | 86 | - | - | - | - | - | (Otterbein et al., 2000) |
| *Trametes pubescens* MB 89 | Lap2 | N | A | 65 | 2.6 | 50-60^a^ | <3.0^a^; 3.0-4.5^b^ | 90^a^ (60 °С) | 14^a^; 470^b^; 72^c^; 360^d^ | (Galhaup et al., 2002) |
| *Trametes trogii*  BAFC 463 | Lacc1 | N | n/a | 38 | - | 50^a^ | 3.4^a^ | - | - | (Levin et al., 2002) |
|  | Lacc2 | N | n/a | 60 | - | 50^a^ | 3.4^a^ | - | - |  |
|  | Lacc3 | R | D | 85-90 | - | 50^a^ | 3.5^a^; 4.0^c^ | 45^a^ (70°С) | 250^a^; 2095^c^ | (Campos et al., 2016) |
| *Trametes trogii* B6J | LacI | N | A | 62 | 4.3 | - | 2.5^a^; 3.0^c^ | - | 50^a^ | (Zouari-Mechichi et al., 2006) |
|  | LacII | N | A | 62 | 4.5 | - | 2.5^a^; 3.0^c^ | - | 33^a^ |  |
| *Trametes trogii 201* | Lcc1  (POXL3) | N | A | 60 | 3.3 | - | 2.5^a^; 3.5^c^ | - | 8.3^a^; 195^c^; 3073^d^ | (Garzillo et al., 2001) |
|  | Lcc1 | R | A | 64 | - | - | 2.5^a^; 3.5^c^ | - | 9.2^a^; 529^c^; 4177^d^ | (Colao et al., 2006) |
| *Trametes vesicolor* ATCC 32745 | LacIIIb | N | A | 59 | 2.75-3.23 | - | 3.6^c^ | - | 50^a^; 275^c^ | (Bertrand et al., 2002; Jolivalt et al., 2005) |
|  | LacIIIb | R | A | 60 | - | - | 3.5-4.0^c^ | - | 26^a^; 235^c^ | (Jolivalt et al., 2005) |
| *Trametes vesicolor* UAMH 8272 | Lac1 | R | A | - | 3.0-4.0 | - | - | - | - | (Fujihiro et al., 2009) |
|  | Lac4 | R | H | - | 3.6 | - | - | - | - |  |
| *Trametes versicolor* 951022 | Lac951022 | N | A | 97 | - | 50^a^ | 3.0-4.0^a^ | 45^a^ (60 °С) | 12.8^a^ | (Han et al., 2005) |
| **Strain** | **Protein** | **Origin** | **Clade** | **Мw, kDa** | ***pI*** | **Topt, °C** | **pHopt** | **Thermostability (τ_1/2_), min** | **К_м_, µМ** | **Ref.** |
| *Trametes versicolor 3086* | Lccβ | R | A | 72 | 3.07-3.27 | 80^a^ | 2.3^a^ | - | 88^a^ | (Cassland and Jonsson, 1999; Jonsson et al., 1962; Koschorreck et al., 2008; Necochea et al., 2005) |
|  | Lccα | R | BE | 72 | 4.64-6.76 | 75^a^ | 1.9^a^ | - | 22^a^ |  |
|  | Lccɣ | R | H | 72 | 4.2 | 60^a^ | 3.1^a^ | - | 359^a^ | (Koschorreck et al., 2008; Necochea et al., 2005) |
|  | Lacδ | R | G | 72 | 4.2 | 45^a^ | 3.1^a^ | - | 2262^a^ |  |
| *Trametes villosa CBS 678.70* | form 1 (Lcc1) | N | A | 60-70 | 3.5 | - | 2.7^a^; 5.0-5.5^f^ | - | - | (Yaver and Golightly, 1996; Yaver et al., 1996) |
|  | form 2 *(*Lcc2*)* | N | BE* | 60-70 | 5-6.8 | - | 6^a^; 5.0-5.5^f^ | - | - |  |
|  | form 3 (Lcc2) | N | BE* | 60-70 | 6.2-6.8 | - | 6^a^; 5.0-5.5^f^ | - | - |  |
| Trametes sp. 420 | LacE | N | A | 62.8 | 4.6 | 50^d^ | 2.7^a^; 4.3^d^ | 78^a^ (60 °С) | 35^a^; 41^c^; 254^d^; 5^f^ | (Tong et al., 2007) |
|  | LacDx | R | C * | 77 | - | 45^d^ | 6.0^d^ | 2^d^ (60 °С) | 427^a^ | (Hong et al., 2007; Zhou et al., 2007) |
|  | LacDe | R | C * | 78 | - | 45^d^ | 6.0^d^ | 2^d^ (60 °С) | 604^a^ |  |
| Trametes sp. C30 (Marasmius quercophilus) | Lac1 | N | A | 63 | 3.6 | 75^f^ | 4.5-5.0 ^f^ | - | 2.9^a^; 71^d^; 0.9^f^ | (Dedeyan et al., 2000; Klonowska et al., 2002) |
|  | Lac2 | N | H | 65 | 3.2 | 55^f^ | 5.5-6.0^f^ | - | 536^a^; 1006^d^; 6.8^f^ | (Klonowska et al., 2002) |
|  | Lac3 | R | C | 75 | - | 55^f^ | 5.5-6.0^f^ | - | 280^a^; 1600^d;^ 14^f^ | (Klonowska et al., 2005) |
| *Trametes ochracea 92-78* | - | N | n/a | 64 | 4.7 | - | 3.7-4.9^b^ | - | 110^b^; 90^d^ | (Shleev et al., 2004) |
| *Coriolus hirsutus* P04 | - | N | n/a | 73 | 7.4 | 45^a^ | 2.5^a^; 4.0^c^ | - | 56.7^a^; 39.9^b^; 53^c^; 10.9^d^ | (Shin and Lee, 2000) |
| *Trametes hirsuta* lg-9 | Laclg9 | N | A | 90 | 4.3 | 85^c^ | 2.4^a^; 2.5^c^ | - | 70^a^; 200^c^ | (Haibo et al., 2009) |
| **Strain** | **Protein** | **Origin** | **Clade** | **Мw, kDa** | ***pI*** | **Topt, °C** | **pHopt** | **Thermostability (τ_1/2_), min** | **К_м_, µМ** | **Ref.** |
| *Trametes hirsuta* Bm2 | LacI | N | n/a* | 65 | 6.4 | 45-60^a^ | 4.5^a^ | - | 0.49^a^ | (Zapata-Castillo et al., 2015) |
|  | LacII | N | n/a* | 65 | 6.9 | 40-60^a^ | 4.5^a^ | - | 0.53^a^ |  |
|  | LacIII | N | n/a* | 65 | 6.6 | 40^a^ | 4.5^a^ | - | 0.41^a^ | (Zapata-Castillo et al., 2015) |
| *Trametes hirsuta* 072 | LacA | N | A | 60 | 3.7 | 55-70^a^; 55^b^; 55-70^c^ | 1.81-3.0^a^; 4.0^b,c^ | 15^a^ (60 °С) | 17^a^; 24^c^, 173^d^ | (Glazunova et al., 2018; Savinova et al., 2017) |
|  | rLacC | R | C | 67 | 3.1 | 65^a^; 60^b,c^ | 1.81-2.25^a^; 5.25^b,c^ | <5^a^ (60 °С) | 534^a^; 589^c^; 15742^d^ | (Savinova et al., 2017) |
|  | rLacD | R | D | 75 | 6.5 | 65-75^a^; 75^b^; 65-75^c^ | 2.25-2.75^a^; 3.75-4.5^b,c^ | 22^a^ (60 °С) | 37^a^; 17^c^; 931^d^ | This study |
|  | rLacF | R | F | 70 | 4.3 | 65-75^a^; 55-70^b^; 50-60^c^ | 2.5-3.5^a^; 5.0^b^; 4.25^c^ | 10^a^ (60 °С) | 88,5^a^; 79^c^; 2766^d^ | This study |

**References S**

Bertrand, T., Jolivalt, C., Briozzo, P., Caminade, E., Joly, N., Madzak, C., Mougin, C., 2002. Crystal structure of a four-copper laccase complexed with an arylamine: Insights into substrate recognition and correlation with kinetics. Biochemistry 41, 7325–7333. https://doi.org/10.1021/bi0201318

Campos, P.A., Levin, L.N., Wirth, S.A., 2016. Heterologous production, characterization and dye decolorization ability of a novel thermostable laccase isoenzyme from *Trametes trogii* BAFC 463. Process Biochem. 51, 895–903. https://doi.org/10.1016/j.procbio.2016.03.015

Cassland, P., Jonsson, L.J., 1999. Characterization of a gene encoding *Trametes versicolor* laccase A and improved heterologous expression in Saccharomyces cerevisiae by decreased cultivation temperature. Appl. Microbiol. Biotechnol. 52, 393–400.

Colao, M.C., Lupino, S., Garzillo, A.M., Buonocore, V., Ruzzi, M., 2006. Heterologous expression of lcc1 gene from *Trametes trogii* in *Pichia pastoris* and characterization of the recombinant enzyme. Microb. Cell Fact. 5, 31. https://doi.org/10.1186/1475-2859-5-31

Dantán-González, E., Vite-Vallejo, O., Martínez-Anaya, C., Méndez-Sánchez, M., González, M.C., Palomares, L.A., Folch-Mallol, J., 2008. Production of two novel laccase isoforms by a thermotolerant strain of *Pycnoporus sanguineus* isolated from an oil-polluted tropical habitat. Int. Microbiol. 11, 163–169. https://doi.org/10.2436/20.1501.01.56

Dedeyan, B., Klonowska, A., Tagger, S., Tron, T., Iacazio, G., Gil, G., Petit, J. Le, 2000. Biochemical and Molecular Characterization of a Laccase from Marasmius quercophilus Biochemical and Molecular Characterization of a Laccase from Marasmius quercophilus. Appl. Environ. Microbiol. 66, 925–929. https://doi.org/10.1128/AEM.66.3.925-929.2000.Updated

Eggert, C., Temp, U., Eriksson, K.E., Eggert, C., Temp, U., 1996. The ligninolytic system of the white rot fungus Pycnoporus cinnabarinus : purification and characterization of the laccase . The Ligninolytic System of the White Rot Fungus *Pycnoporus cinnabarinus* : Purification and Characterization of the Laccase 62, 1151–1158.

Fujihiro, S., Higuchi, R., Hisamatsu, S., Sonoki, S., 2009. Metabolism of hydroxylated PCB congeners by cloned laccase isoforms. Appl. Microbiol. Biotechnol. 82, 853–860. https://doi.org/10.1007/s00253-008-1798-2

Galhaup, C., Goller, S., Peterbauer, C.K., Strauss, J., Haltrich, D., 2002. Characterization of the major laccase isoenzyme from *Trametes pubescens* and regulation of its synthesis by metal ions. Microbiology 148, 2159–2169. https://doi.org/10.1099/00221287-148-7-2159

Garzillo, A.M., Colao, M.C., Buonocore, V., Oliva, R., Falcigno, L., Saviano, M., Santoro, A.M., Zappala, R., Bonomo, R. Pietro, Bianco, C., Giardina, P., Palmieri, G., Sannia, G., 2001. Structural and kinetic characterization of native laccases from *Pleurotus ostreatus*, *Rigidoporus lignosus*, and *Trametes trogii*. J. Protein Chem. 20, 191–201. https://doi.org/10.1023/A:1010954812955

Glazunova, O.A., Shakhova, N.V., Psurtseva, N.V., Moiseenko, K.V., Kleimenov, S.Y., Fedorova, T.V., 2018. White-rot basidiomycetes *Junghuhnia nitida* and *Steccherinum bourdotii*: Oxidative potential and laccase properties in comparison with *Trametes hirsuta* and *Coriolopsis caperata*. PLoS One 13, e0197667. https://doi.org/10.1371/journal.pone.0197667

Haibo, Z., Yinglong, Z., Feng, H., Peiji, G., Jiachuan, C., 2009. Purification and characterization of a thermostable laccase with unique oxidative characteristics from *Trametes hirsuta*. Biotechnol. Lett. 31, 837–843. https://doi.org/10.1007/s10529-009-9945-0

Han, M.-J.M.-J., Choi, H.-T., Song, H.-G., Han, M.-J.M.-J., Choi, H.-T., Song, H.-G., 2005. Purification and characterization of laccase from the white rot fungus *Trametes versicolor*. J. Microbiol. 43, 555–60. https://doi.org/10.1016/0922-338X(95)98183-L

Hong, Y.Z., Zhou, H.M., Tu, X.M., Li, J.F., Xiao, Y.Z., 2007. Cloning of a laccase gene from a novel basidiomycete *Trametes* sp. 420 and its heterologous expression in *Pichia pastoris*. Curr. Microbiol. 54, 260–265. https://doi.org/10.1007/s00284-006-0068-8

Jolivalt, C., Madzak, C., Brault, A., Caminade, E., Malosse, C., Mougin, C., 2005. Expression of laccase IIIb from the white-rot fungus *Trametes versicolor* in the yeast *Yarrowia lipolytica* for environmental applications. Appl. Microbiol. Biotechnol. 66, 450–456. https://doi.org/10.1007/s00253-004-1717-0

Jonsson, M., Pettersson, E., Reinhammar, B., 1962. The Isoelectric Spectra of Fungal Laccase A and B. Acta Chem. Scand. 22, 2135–2140.

Kim, H., Lee, S., Ryu, S., Choi, H.T., 2012. Decolorization of remazol brilliant blue R by a purified laccase of polyporus brumalis. Appl. Biochem. Biotechnol. 166, 159–164. https://doi.org/10.1007/s12010-011-9412-y

Klonowska, A., Gaudin, C., Asso, M., Fournel, A., Réglier, M., Tron, T., 2005. LAC3, a new low redox potential laccase from *Trametes* sp. strain C30 obtained as a recombinant protein in yeast. Enzyme Microb. Technol. 36, 34–41. https://doi.org/10.1016/j.enzmictec.2004.03.022

Klonowska, A., Gaudin, C., Fournel, A., Asso, M., Le Petit, J., Giorgi, M., Tron, T., 2002. Characterization of a low redox potential laccase from the basidiomycete c30. Eur. J. Biochem. 269, 6119–6125. https://doi.org/10.1046/j.1432-1033.2002.03324.x

Koschorreck, K., Richter, S.M., Swierczek, A., Beifuss, U., Schmid, R.D., Urlacher, V.B., 2008. Comparative characterization of four laccases from *Trametes versicolor* concerning phenolic C-C coupling and oxidation of PAHs. Arch. Biochem. Biophys. 474, 213–219. https://doi.org/10.1016/j.abb.2008.03.009

Levin, L., Forchiassin, F., Ramos, A.M., 2002. Copper induction of lignin-modifying enzymes in the white-rot fungus *Trametes trogii*. Mycologia 94, 377–383. https://doi.org/10.1080/15572536.2003.11833202

Nakade, K., Nakagawa, Y., Yano, A., Sato, T., Sakamoto, Y., 2010. Characterization of an extracellular laccase, PbLac1, purified from Polyporus brumalis. Fungal Biol. 114, 609–618. https://doi.org/10.1016/j.funbio.2010.05.002

Necochea, R., Valderrama, B., Díaz-Sandoval, S., Folch-Mallol, J.L., Vázquez-Duhalt, R., Iturriaga, G., 2005. Phylogenetic and biochemical characterisation of a recombinant laccase from *Trametes versicolor*. FEMS Microbiol. Lett. 244, 235–241. https://doi.org/10.1016/j.femsle.2005.01.054

Oda, Y., Adachi, K., Aita, I., Ito, M., 1991. Excreted by Pycnoporus coccineus 55, 1393–1395.

Otterbein, L., Record, E., Chereau, D., Herpoël, I., Asther, M., Moukha, S.M., 2000. Isolation of a new laccase isoform from the white-rot fungi *Pycnoporus cinnabarinus* strain ss3. Can. J. Microbiol. 46, 759–763.

Ramírez-Cavazos, L.I., Junghanns, C., Ornelas-Soto, N., Cárdenas-Chávez, D.L., Hernández-Luna, C., Demarche, P., Enaud, E., García-Morales, R., Agathos, S.N., Parra, R., 2014. Purification and characterization of two thermostable laccases from *Pycnoporus sanguineus* and potential role in degradation of endocrine disrupting chemicals. J. Mol. Catal. B Enzym. 108, 32–42. https://doi.org/10.1016/j.molcatb.2014.06.006

Record, E., Punt, P.J., Chamkha, M., Labat, M., Van Den Hondel, C.A.M.J.J., Asther, M., 2002. Expression of the *Pycnoporus cinnabarinus* laccase gene in Aspergillus niger and characterization of the recombinant enzyme. Eur. J. Biochem. 269, 602–609. https://doi.org/10.1046/j.0014-2956.2001.02690.x

Ryu, S.H., Lee, A.Y., Kim, M., 2008. Molecular characteristics of two laccase from the basidiomycete fungus Polyporus brumalis. J. Microbiol. 46, 62–69. https://doi.org/10.1007/s12275-007-0110-y

Savinova, O.S., Moiseenko, K. V., Vavilova, E.A., Tyazhelova, T. V., Vasina, D. V., 2017. Properties of two laccases from the *Trametes hirsuta* 072 multigene family: Twins with different faces. Biochimie 142, 183–190. https://doi.org/10.1016/j.biochi.2017.09.013

Shin, K.S., Lee, Y.J., 2000. Purification and characterization of a new member of the laccase family from the white-rot basidiomycete *Coriolus hirsutus*. Arch. Biochem. Biophys. 384, 109–115. https://doi.org/10.1006/abbi.2000.2083

Shinagawa, E., 1995. Purification and Characterization of Lactase from White Rot Fungus. J. Ferment. Bioeng. 80, 91–93. https://doi.org/10.1016/0922-338X(95)98183-L

Shleev, S. V., Morozova, O. V., Nikitina, O. V., Gorshina, E.S., Rusinova, T. V., Serezhenkov, V.A., Burbaev, D.S., Gazaryan, I.G., Yaropolov, A.I., 2004. Comparison of physico-chemical characteristics of four laccases from different basidiomycetes. Biochimie 86, 693–703. https://doi.org/10.1016/j.biochi.2004.08.005

Temp, U., Zierold, U., Eggert, C., 1999. Cloning and characterization of a second laccase gene from the lignin-degrading basidiomycete *Pycnoporus cinnabarinus*. Gene 236, 169–177. https://doi.org/10.1016/S0378-1119(99)00239-5

Tong, P., Hong, Y., Xiao, Y., Zhang, M., Tu, X., Cui, T., 2007. High production of laccase by a new basidiomycete, *Trametes* sp. Biotechnol. Lett. 29, 295–301. https://doi.org/10.1007/s10529-006-9241-1

Uzan, E., Nousiainen, P., Balland, V., Sipila, J., Piumi, F., Navarro, D., Asther, M., Record, E., Lomascolo, A., 2010. High redox potential laccases from the ligninolytic fungi *Pycnoporus coccineus* and *Pycnoporus sanguineus* suitable for white biotechnology: From gene cloning to enzyme characterization and applications. J. Appl. Microbiol. 108, 2199–2213. https://doi.org/10.1111/j.1365-2672.2009.04623.x

Xiao, Y.Z., Chen, Q., Hang, J., Shi, Y.Y., Wu, J., Hong, Y.Z., Wang, Y.P., 2004. Selective induction, purification and characterization of a laccase isozyme from the basidiomycete *Trametes* sp. AH28-2. Mycologia 96, 26–35. https://doi.org/10.1080/15572536.2005.11832993

Xiao, Y.Z., Tu, X.M., Wang, J., Zhang, · M, Cheng, Q., Zeng, W.Y., Shi, Y.Y., 2003. Purification, molecular characterization and reactivity with aromatic compounds of a laccase from basidiomycete *Trametes* sp. strain AH28-2. Appl Microbiol Biotechnol 60, 700–707. https://doi.org/10.1007/s00253-002-1169-3

Yaver, D.S., Golightly, E.J., 1996. Cloning and characterization of three laccase genes from the white-rot basidiomycete *Trametes villosa*: Genomic organization of the laccase gene family. Gene 181, 95–102. https://doi.org/10.1016/S0378-1119(96)00480-5

Yaver, D.S., Xu, F., Golightly, E.J., Brown, K.M., Brown, S.H., Rey, M.W., Schneider, P., Halkier, T., Mondorf, K., Dalboge, H., Yaver, D.S., Xu, F., Golightly, E.J., Brown, K.I.M.M., Brown, S.H., Rey, M.W., Schneider, P., Halkier, T., Mondorf, K., 1996. cloning , and expression of two laccase genes from the white rot basidiomycete *Trametes villosa* . Purification , Characterization , Molecular Cloning , and Expression of Two Laccase Genes from the White Rot Basidiomycete *Trametes villosa*. Appl. Environ. Microbiol. 62, 834–841.

Zapata-Castillo, P., Villalonga-Santana, L., Islas-Flores, I., Rivera-Muñoz, G., Ancona-Escalante, W., Solís-Pereira, S., 2015. Synergistic action of laccases from *Trametes hirsuta* Bm2 improves decolourization of indigo carmine. Lett. Appl. Microbiol. 61, 252–258. https://doi.org/10.1111/lam.12451

Zhang, H., Hong, Y.Z., Xiao, Y.Z., Yuan, J., Tu, X.M., Zhang, X.Q., 2006. Efficient production of laccases by *Trametes* sp. AH28-2 in cocultivation with a *Trichoderma strain*. Appl. Microbiol. Biotechnol. 73, 89–94. https://doi.org/10.1007/s00253-006-0430-6

Zhou, H.M., Hong, Y.Z., Xiao, Y.Z., Cui, T.J., Wang, X.T., Pu, C.L., 2007. High Output of a *Trametes Laccase* in *Pichia pastoris* and Characterization of Recombinant Enzymes. Chin. J. Biotechnol. 23, 1055–1059. https://doi.org/10.1016/S1872-2075(07)60063-6

Zouari-Mechichi, H., Mechichi, T., Dhouib, A., Sayadi, S., Martínez, A.T., Martínez, M.J., 2006. Laccase purification and characterization from Trametes trogii isolated in Tunisia: decolorization of textile dyes by the purified enzyme. Enzyme Microb. Technol. 39, 141–148. https://doi.org/10.1016/j.enzmictec.2005.11.027
